# (Iso)quinoline-Modified Aza-Boron-Dipyrromethenes Near-Infrared-II Fluorescence/Photoacoustic Nanotheranostics for Cervical Tumor Photothermal Therapy

**DOI:** 10.34133/bmr.0298

**Published:** 2025-12-09

**Authors:** Kexin Wang, Zhen Wang, Jianfeng Qiu, Yunjian Xu

**Affiliations:** ^1^ School of Radiology, Shandong First Medical University and Shandong Academy of Medical Sciences, Taian 271000, China.; ^2^ Medical Science and Technology Innovation Center, Shandong First Medical University and Shandong Academy of Medical Sciences, Jinan 250117, China.

## Abstract

NIR-II small-molecule-based bimodal imaging systems accurately unify diagnosis and therapeutics for precision tumor therapy, which is attributed to their easily modifiable structures, high potential biocompatibility. In particular, the highly efficient photodiagnostic agent with high light-to-heat transformation performance and fluorescence/photoacoustic imaging (FLI/PAI) with the range of near-infrared-II (NIR-II; 900 to 1,700 nm) has emerged as a popular research topic. This study reported a series of Aza-boron-dipyrromethenes (Aza-BODIPY) dyes (Aza-A/B/C) with donor–acceptor structure through the introduction of diethylaminobenzene (electron donor) and (iso)quinoline (electron acceptor) into the Aza-BODIPY backbone. Compared to Aza-A/B, the enhanced light trapping ability, the decreased NIR-II fluorescence emission performance, and poor reactive oxygen species generation capacity made Aza-C as an optimal photothermal agent. Through 1,2-distearoyl-sn-glycero-3-phosphoethanolamine-N-[methoxy(polyethylene glycol)-2000] (DSPE-mPEG_2000_) capping, the as-prepared Aza-C nanoparticles (Aza-C NPs) showed excellent biocompatibility, super stability, outstanding light-to-heat transformation performance (ƞ = 58.2%), as well as concentration-dependent linear FL/PA signals, which guaranteed that Aza-C NPs could be successfully utilized for NIR-II FLI/PAI-directed efficient photothermal therapy (PTT) of cervical tumor, with high tumor inhibition rates of over 90%. Introducing diethylaminobenzene and (iso)quinoline to Aza-BODIPY backbone help to construct NIR-II Aza-C dye for NIR-II FLI/PAI-directed efficient tumor PTT. This novel approach offers a promising avenue toward the ablation of tumors in deep tissues.

## Introduction

Cervical tumor, ranked as the second most common and the third most lethal female malignancy, remains one of the most common and fatal gynecologic malignancies worldwide [1,2]. In the face of the clinical challenges posed by the high recurrence and metastasis rates of cervical tumors, the development of novel diagnostic and therapeutic strategies is particularly urgent [3–5]. Currently, near-infrared-I (NIR-I), especially NIR-II (900 to 1,700 nm), region imaging-directed tumor therapy has been extensively explored because of their satisfactory tissue imaging penetration depth. NIR-II fluorescence/photoacoustic imaging (FLI/PAI)-directed tumor diagnosis and photothermal therapy (PTT) is a novel comprehensive technology [6–8]. NIR-II FLI can remarkably improve imaging sensitivity [9–11]. In addition, NIR-II PAI has the advantage of deep penetration [12–14], and their complementary advantages provide key technical support for accurate tumor localization. In the therapeutic aspect, tumor ablation is achieved by releasing heat through light irradiation of the diagnostic agent. Therefore, the construction of NIR-II FLI/PAI-based photothermal diagnostic agent can make full use of the complementary advantages of FLI/PAI to achieve efficient tumor PTT in deep tissue [15–17].

A large number of diagnostic agents such as rare-earth-doped nanoparticles (NPs) and transition metal oxysulfides have been found to be NIR-II FLI/PAI-directed efficient PTT in recent years [18], but their potential biotoxicity and nondegradability severely limit their clinical applications [19]. In contrast, diagnostic agents based on organic NPs, especially small-molecule systems, are gradually becoming a research hotspot by virtue of their tunable metabolic pathways and promising excellent biocompatibility [20–22]. Among them, compared to boron-dipyrromethenes (BODIPY) dyes with the same skeleton, Aza-BODIPY showed obviously red-shifted NIR absorption/emission, indicating their matching biological applications [23–25]. Besides, compared to the cyanine dyes, Aza-BODIPY also exhibited superior anti-photobleaching property, which played an extremely important role in imaging-directed photoinduced tumor treatment [26,27]. In addition, few works have verified that Aza-BODIPY dyes as natural photothermal agent and PAI contrast could fulfill NIR-II FLI and efficient tumor PTT through rational design, indicating their potential for realizing NIR-II FLI/ PAI-directed efficient tumor PTT [28].

In this study, a series of NIR-II fluorescent dyes (Aza-A/B/C) with donor–acceptor (D–A) structure were designed and synthesized based on the Aza-BODIPY backbone by the combination of diethylaminobenzene as a strong electron donor and (iso)quinolines as an electron acceptor. The systematic photophysical exploration confirmed that Aza-C with optimal NIR absorption accompanying poor NIR-II emission and reactive oxygen species (ROS) production ability showed super heat production performance. The self-assembly of DSPE-mPEG_2000_ and Aza-C formed uniformly distributed Aza-C NPs with excellent heat production performance, NIR-II FL/PA signals, as well as low cellular dark toxicity, which have enabled its effective use for NIR-II FLI/PAI-directed PTT of cervical tumors.

## Materials and Methods

### Materials

Photothermal effect of Aza-C NPs: The photothermal performance of Aza-C NPs was evaluated by monitoring temperature changes in aqueous dispersion using infrared thermography (InfiRa AT61U). Solutions (300 μl) at varying concentrations or under different optical power densities were exposed to 808-nm light, and their thermal profiles were recorded. To assess photothermal stability, the temperature kinetics of a 20 μM Aza-C NP solution were measured over multiple heating (light-on) and cooling (light-off) cycles. The light-to-heat transformation efficiency of Aza-C NPs was calculated based on established methodologies, incorporating the observed temperature dynamics and light parameters.

### FL/PA signals of Aza-C NPs

The FL/PA signals of Aza-C NPs exhibit a pronounced concentration-dependent behavior. The fluorescence signals of Aza-C NP solutions with different concentrations of Aza-C NPs were obtained using a NIR FLI system (emission window λ_em_ > 980 nm). The PA signal was acquired from different concentrations of Aza-C NP solutions using a PAI system (λ_ex_ = 910 nm).

### Photothermal generation capacity of Aza-C NPs

In order to investigate the photothermal properties of Aza-C NPs, the temperature changes of Aza-C NPs (20 μM) exposed to 808-nm light using different power densities (0.2 to 0.8 W cm^−2^) and different concentrations (5 to 40 μM) of Aza-C NPs exposed to 808-nm light (0.6 W cm^−2^ for 10 min) were recorded by infrared thermography. To assess photothermal stability, 5 consecutive heating and cooling cycles were performed. Each cycle comprised 10 min of light exposed to (808 nm, 0.6 W cm^−2^) followed by natural cooling to ambient temperature.

### Cytotoxicity assay

The mouse breast cancer cell line (4T1), human cervical cancer cell line (Hela), and mouse embryonic fibroblasts (NIH3T3) were obtained from Wuhan Shane Biotechnology Co. and maintained in complete medium at 37 °C in a 5% CO₂ atmosphere. To evaluate the dark toxicity and phototoxicity of Aza-C NPs, cell viability was assessed using the Cell Counting Kit-8 (CCK-8) assay. Briefly, Hela, 4T1, and NIH3T3 cells were treated with varying concentrations of Aza-C NPs for 24 h. For the phototoxicity study, cells were subsequently exposed to an 808-nm light (0 to 0.8 W cm^−2^, 10 min). After treatment, each well received 10 μl of CCK-8 reagent (5 mg ml^−1^) and was maintained at normal temperatures for 2 h. Cellular proliferation was subsequently quantified by detecting the 450-nm (OD₄₅₀) wavelength absorption.

### In vitro photothermal toxicity of Aza-C NPs

Cell viability was assessed using calcein-AM/propidium iodide (PI) dual-labeling, where viable cells (calcein-AM-positive) and nonviable cells (PI-positive) were quantified by both confocal microscopy and flow cytometry.

### Animals and tumor model

Female Balb/c-nu nude mice (about 20 g, 5 to 7 weeks old) were obtained from the Animal Experiment Center of Jiangsu Huachuang Xinnuo Pharmaceutical Science and Technology Co. Ltd. [license no.: SCXK (Su) 2020-0009]. All procedures involving animals were performed in compliance with institutional animal care protocols and were approved by the Institutional Animal Care and Use Committee (IACUC) of Shandong First Medical University (approval no.: W202405300471).

### In vivo photothermal assessment

All animal imaging and therapeutic interventions were conducted in accordance with approved protocols for animal research. Hela nude mice (tumor volume ≈ 100 mm^3^) received intravenously administered Aza-C NPs (120 μl, 200 μM) and subjected to photothermal imaging under 808-nm light irradiation (0.6 W cm^−2^, 10 min) at predetermined time points.

### In vivo photothermal efficacy

Tumor dimensions were measured using digital calipers, and volumes (*V*) were calculated using the ellipsoid formula *V* = (*L* × *W*^2^)/2, where *L* and *W* represent the major and minor tumor axes, respectively. Throughout the 21-d study period, body weight and tumor progression were assessed at 2-d intervals. Mice were considered as ethically dead when the tumor length was more than 15 mm, or when cachexia or ulceration occurred [29]. Finally, the major organs (liver, kidney, etc.) were collected for histopathological analysis, and the blood samples were collected for systemic safety assessment.

## Results

### Design and preparation of Aza-A/B/C dyes

The electronic D–A type structure based on the Aza-BODIPY core showed satisfactory NIR absorption/emission; especially, the alkylaniline structure as the electronic donor in Aza-BODIPY could fulfill almost NIR-II emission [30]. Besides, (iso)quinoline with large conjugate skeleton accompanying electron-absorbing ability helps to enhance light capture ability and red-shifted absorption and emission of luminophore [31]. With the above consideration, a series of Aza-BODIPY (Aza-A/B/C) dyes were constructed by introducing diethylaminobenzene and (iso)quinoline to realize their NIR-II FLI/PAI-directed efficient tumor PTT. In Fig. S1, the synthetic route and molecular structure of Aza-A/B/C is shown. Aza-A/B/C could be prepared by 4 steps, referring to previous works [30]. In contrast to the challenges associated with preparing Aza-BODIPY dyes modified with electron-withdrawing functional groups, which often require an additional step of reaction before forming Aza-DIPY skeleton [32], it could be resolved by high temperature in regular corresponding reaction steps in this work. Finally, the corresponding media products and Aza-A/B/C dyes were obtained and confirmed by ^1^H nuclear magnetic resonance (Figs. S2 to S4).

### Photophysical properties of Aza-A/B/C dyes

**Fig. 1.**
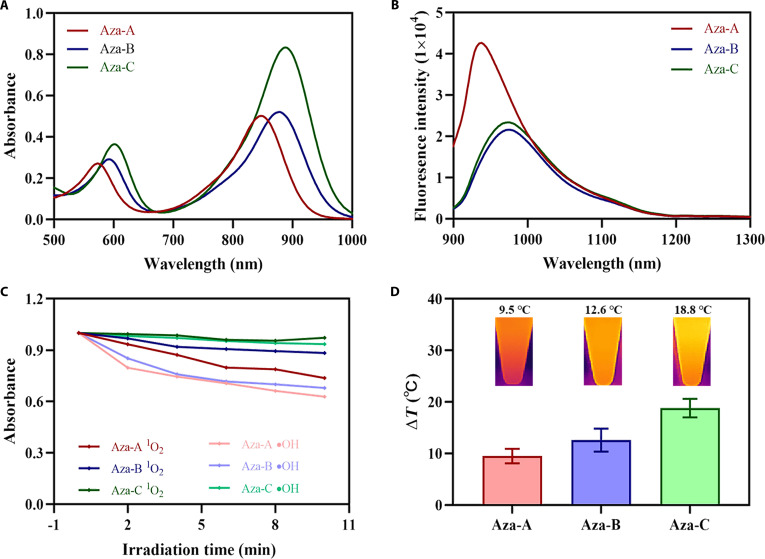
Photophysical properties of Aza-A/B/C dyes. (A) Absorption and (B) emission spectra of Aza-A/B/C in DMF solutions (10 μM). (C) ROS generation capability of Aza-A/B/C dyes. (D) Comparison of heat production performance of Aza-A/B/C dyes.

The photophysical properties of Aza-A/B/C in *N*,*N*-dimethylformamide (DMF) solution were first systematically characterized. The maximum absorption peaks of Aza-A/B/C were around 846, 878, and 888 nm, respectively (Fig. 1A), and the corresponding molar absorption coefficients (ɛ) were 5.0 × 10^4^, 5.2 × 10^4^, and 8.3 × 10^4^ l mol^−1^ cm^−1^, respectively. Aza-A/B/C showed intense emission in the NIR-II range with emission peaks at 946, 990, and 990 nm, which is in line with the narrow energy gaps (Δ*E*_S1-S0_) of 1.88, 1.87, and 1.80 eV, respectively, calculated through density functional theory (Fig. 1B and Figs. S5 and S6) [33]. Besides, under the same excited condition and concentration, compared to Aza-A/B, Aza-C showed an increased NIR-II absorption and a decreased NIR-II emission with poor luminescence quantum yield (Φ_Aza-A_/3, Φ_Aza-B_/2), suggesting its potential superior thermogenic performance. To further demonstrate the enhanced thermogenic potential of Aza-C dye, the ROS-generating capacity and photothermal properties of the Aza-A/B/C dyes were further investigated. As shown in Fig. 1C, compared to those of Aza-A/B, Aza-C showed poor or almost no ^1^O_2_ and hydroxyl radical generation performance. According to the Jablonski energy diagram, it is known that the excited electrons of Aza-C hardly undergo intersystem crossing process. This means that the energy released by the quenching of most excited state electrons of the Aza-C dye are almost utilized for heat generation. Then, the photothermal performance test of Aza-A/B/C dyes (20 μM) in DMF under irradiation (0.6 W cm^−2^, 10 min) was explored, referring to previous works [34]. As shown in Fig. 1D, Aza-A/B showed a temperature increase of 9.5 and 12.6 °C, respectively, which is obviously lower than that of Aza-C (18.8 °C), which matched well with the fact that Aza-C showed increased NIR-II absorption as well as decreased NIR-II emission and poor ROS production performance. Therefore, the Aza-C dye with the optimal heat production performance was selected for subsequent experiments.

### Photophysical properties of Aza-C NPs

**Fig. 2.**
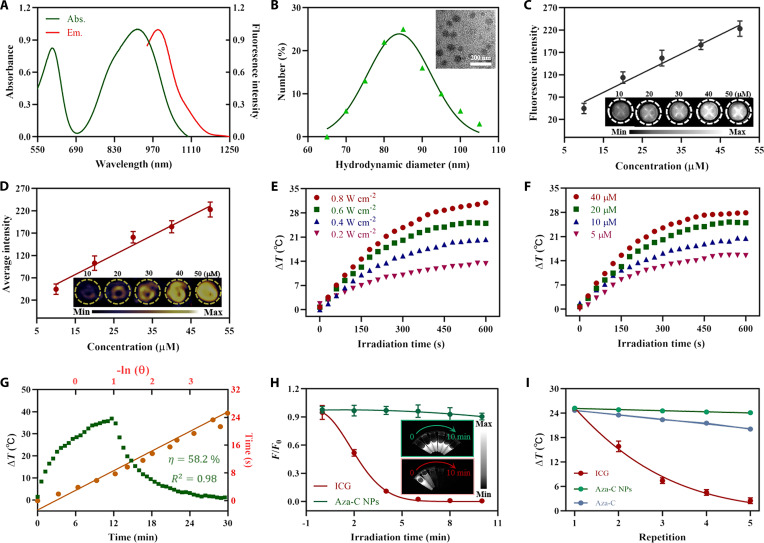
Photophysical properties of Aza-C NPs. (A) Normalized absorption and fluorescence spectra of Aza-C NPs in PBS. (B) Hydrodynamic size of Aza-C NPs in PBS. Inset: TEM image of Aza-C NPs. Scale bar, 200 nm. (C) Quantitative relationship between NIR-II fluorescence intensity and Aza-C NP concentration (λ_em_ > 980 nm). Inset: NIR-II FLI photos of Aza-C NPs at different concentrations. (D) Quantitative relationship between photoacoustic intensity and Aza-C NP concentration (λ_ex_ = 910 nm). Inset: NIR-II PAI photos of Aza-C NPs at different concentrations. (E) Photothermal conversion of Aza-C NPs (20 μM) at different light power densities. (F) Photothermal conversion of different concentrations of Aza-C NPs at 0.6 W cm^−2^ light power density. (G) Photothermal conversion efficiency of Aza-C NPs. (H) NIR-II relative fluorescence intensity changes of Aza-C NPs (20 μM, 0.6 W cm^−2^) and ICG under 808-nm light irradiation (0 to 10 min). (I) Photothermal stability of Aza-C, Aza-C NPs, and ICG in 5 heating–cooling cycles under 808-nm light irradiation (0.6 W cm^−2^).

In order to enhance the biocompatibility of Aza-C, DSPE-mPEG_2000_ and Aza-C were utilized to self-assemble, constructing uniformly distributed Aza-C NPs. The concentration of the Aza-C NP primary solution was measured as 232 μM through the concentration-dependent standard curve (Fig. S7). Figure 2A shows the absorption and emission spectra of Aza-C NPs in phosphate-buffered saline (PBS) (pH 7.4), with its maximum absorption peak located at 911 nm, indicating its promising NIR-II PAI performance, and the emission spectrum concentrated at 900 to 1,150 nm, revealing its potential application in NIR-II FLI. As shown in Fig. 2B, transmission electron microscopy (TEM) measurements showed that the Aza-C NPs had a spherical structure, with a size of 68 nm. Dynamic light scattering (DLS) further confirmed that the mean hydrodynamic size of Aza-C NPs was 86 nm, which is larger than the size observed by TEM, which is attributed to the swelling of the micelles in the aqueous system, and the large size of the Aza-C NPs contributes to their accumulation in tumor tissue through the enhanced permeability and retention (EPR) effect [35]. Given the excellent NIR absorption and emission properties of Aza-C NPs in the NIR-II range, we investigated their concentration-dependent FL and PA signal properties. As shown in Fig. 2C and D, the FL and PA signal intensities of the Aza-C NPs exhibited a good linear enhancement trend as the concentration increased. These were fully consistent with the photos of FL/PA signal enhancement results shown in the inset, which demonstrate the obvious potential of the Aza-C NPs for the following NIR-II FLI/PAI in vivo. Inspired by the excellent heat production performance of Aza-C, the systematic photothermal performance characterization of Aza-C NPs was then launched to quantitatively analyze the light-to-heat transformation properties. After 10 min of irradiation, the temperatures of Aza-C NPs (20 μM) were 13.3, 20.3, 25.0, and 31.0 °C, where the light power density increased from 0.2 to 0.8 W cm^−2^ (Fig. 2E), indicating that the photothermal generating capacity of Aza-C NPs matched well with the increasing light power density. With the given light power density (0.6 W cm^−2^), Aza-C NP solution also showed concentration-dependent (5 to 40 μM) temperature increases of 15.5, 20.5, 25.0, and 27.9 °C (Fig. 2F). These experimental results quantitatively confirmed the marked impact of key parameters such as light power density, Aza-C NP concentration, and irradiation time on photothermal generation ability and fully demonstrated the highly controllable photothermal performance of Aza-C NPs. Especially, Aza-C NP solution (20 μM) showed a 25.0 ± 0.5 °C increase after irradiation (0.6 W cm^−2^) for 10 min, which could ensure effective tumor cell killing while avoiding damage to normal tissue caused by excessive temperature (Fig. 2G) [36]. The light-to-heat transformation efficiency of Aza-C NPs was found to be 58.2% (Fig. 2G and Fig. S8), which verifies its super photothermal effect [37]. This is obviously better than that of most reported photothermal diagnostic agents (Table S1) [20–22,27]. The stability assessment of Aza-C NPs is crucial for their potential biomedical applications. Their structural stability was first evaluated by monitoring their state, DLS, maximum absorption value, and zeta potential over a 20-d period. As shown in Fig. S9A to C, the Aza-C NP solution remained clear and transparent in PBS (pH 7.4). DLS measurements indicated that the particle size remained stable at approximately 85 nm. The maximum absorption value and zeta potential of Aza-C NP solution showed no obvious change. These results collectively demonstrate that Aza-C NPs possess excellent structural stability. The anti-photobleaching resistance of Aza-C NPs was then explored by comparing the NIR-II emission of Aza-C NPs and indocyanine green (ICG). The fluorescence of ICG was almost completely eliminated after 5 min of irradiation, and even after 10 min of irradiation, Aza-C NPs did not undergo photoquenching (Fig. 2H), which was in line with unchanged absorption (Fig. S9D), revealing that Aza-C NPs had super anti-photobleaching property over the commonly used clinical dye ICG. Furthermore, thermal cycling stability tests were then performed on Aza-C, Aza-C NPs, and ICG (Fig. 2I). After 5 irradiation–cooling cycles, the Aza-C NPs retained their capacity for high photothermal generation. In contrast, the photothermal generation of ICG decreased by 36% after the second cycle, and after 5 cycles, only 10% of the heat generation capacity of ICG remained compared with that of the first cycle. The findings indicate that Aza-C NPs exhibit remarkable photothermal generation capacity and stability, along with notable anti-photobleaching properties. These characteristics underscore their potential to serve as high-performance contrast and diagnostic agents for the following biological applications.

### Cytotoxicity studies in vitro

**Fig. 3.**
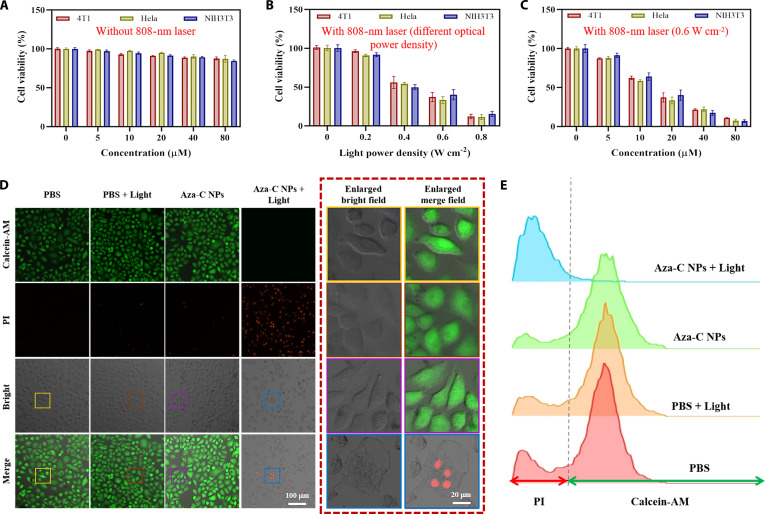
Photothermal properties of Aza-C NPs in cells. (A) Cell viability after incubation with different concentrations of Aza-C NPs for 24 h in various cell lines. (B) Cell viability after laser irradiation at various power densities following 24-h incubation of different cell lines with Aza-C NPs (20 μM). (C) Cell viability after treatment with different concentrations of Aza-C NPs and light irradiation (808 nm, 0.6 W cm^−2^, 10 min) in various cell lines. (D) Confocal FLI of Hela cells stained with calcein-AM (green, live cells) and PI (red, dead cells) after different treatments. Scale bars, 100 μm. The dashed boxes provide magnified views of the corresponding regions of interest from the left image, with a scale bar of 20 μm. (E) Apoptosis analysis of Hela cells with various treatments.

In order to systematically evaluate the PTT effects of Aza-C NPs to tumor cells, common cells in the laboratory [Hela (human cervical tumor), 4T1 (mouse mammary tumor), or NIH3T3 (mouse embryonic fibroblasts) cells] were selected to launch cell viability assays (CCK-8), live/dead cell detection (calcein-AM/PI), and flow cytometry. Firstly, the dark toxicity of Aza-C NPs on Hela, 4T1, and NIH3T3 cells was assessed using the CCK-8 kit. As the concentration of Aza-C NPs increased, no marked change in cell viability was observed. The survival rate of all these cells remained above 89%, even at a high concentration of 80 μM (Fig. 3A), suggesting that Aza-C NPs exhibit very low dark toxicity to these cells. As the power density of laser irradiation increased, the viability of Hela, 4T1, and NIH3T3 cells decreased to varying degrees. At a laser power density of 0.6 W cm^−2^, the cell viabilities of Hela, 4T1, and NIH3T3 treated with Aza-C NPs (20 μM) were 33.4%, 37.2%, and 40.2%, respectively (Fig. 3B). This experiment confirmed the excellent photocontrol cytotoxic properties of Aza-C NPs on Hela, 4T1, and NIH3T3 cells. Besides, with 808-nm light irradiation (0.6 W cm^−2^, 10 min), the Hela, 4T1, and NIH3T3 cell survival rates decreased from 87.9%, 87.3%, and 91.3% to 7.5%, 10.9%, and 6.9% when the cell concentration was increased from 5 to 80 μM, respectively (Fig. 3C). These results indicate that Aza-C NPs have marked concentration-dependent cytotoxicity. Subsequently, we evaluated the cellular uptake of Aza-C NPs. As shown in Fig. S10, no obvious fluorescence signal was detected in untreated Hela cells over 6-h period. In contrast, Hela cells treated with Aza-C NPs exhibited a time-dependent increase in NIR-II fluorescence intensity. Cellular uptake of Aza-C NPs increased progressively during the first 4 h of incubation and reached a plateau after 4 h. Next, Hela cells were co-incubated with 20 μM Aza-C NPs for 4 h, and the therapeutic effect was explored through dual staining experiments using calcein-AM and PI. As shown in Fig. 3D and Fig. S11, Hela and NIH3T3 cells exhibit strong green fluorescence (live cells) in a non-irradiated environment. After 10 min of 808-nm light irradiation (0.6 W cm^−2^), almost all Hela and NIH3T3 cells showed bright red fluorescence (dead cells) accompanying almost no green fluorescence. These results were highly consistent with those of the CCK-8 experiments, which once again proved that Aza-C NPs had excellent PTT effects on Hela and NIH3T3 cells. Furthermore, in order to further quantitatively analyze the PTT effect of Aza-C NPs on Hela cells, an experiment was conducted using flow cytometry, with the same condition as the above. The apoptosis rate of Hela cells treated with Aza-C NPs combined with light irradiation was more than 90%, which was obviously higher than that of the other groups (Fig. 3E). These results collectively confirm that Aza-C NPs have excellent biological safety and good photocontrollable cytotoxicity and can efficiently eliminate tumor cells when activated by a NIR light, providing a solid foundation for subsequent in vivo antitumor studies.

### The distribution of Aza-C NPs and antitumor therapy in vivo

**Fig. 4.**
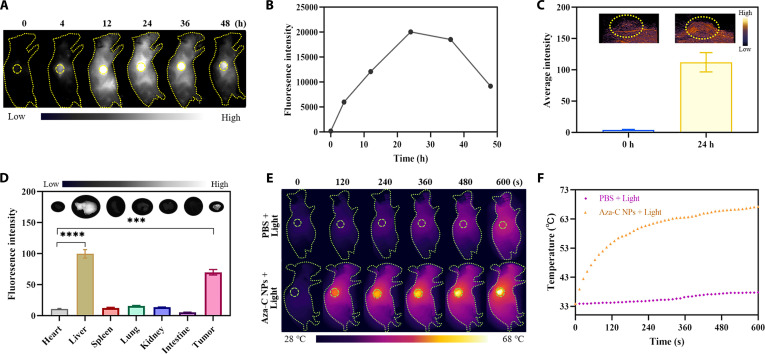
Pharmacokinetics of Aza-C NPs in a Balb/c-nu mice model. (A) NIR-II fluorescence imaging of the tumor region at different time points and (B) fluorescence intensity curves of the tumor region over time. (C) NIR-II PAI of tumor regions before and after intravenous injection of Aza-C NPs into Hela tumor-bearing Balb/c-nu mice. (D) In vitro fluorescence intensity of major organs and tumors 24 h after intravenous injection of Aza-C NPs. (E) Infrared thermal images of mice and (F) photothermal curves of tumor parts after the injection of Aza-C NPs or PBS under 808-nm light irradiation (0.6 W cm^−2^) at different time points. ***P < 0.001, ****P < 0.0001.

In order to launch the following PTT effect of Aza-C NPs on Balb/c-nu mouse tumors, in vivo NIR-II FLI/PAI was carried out via injection of Aza-C NPs into Balb/c-nu mice through the tail vein. The NIR-II fluorescence signal and quantitative intensity at tumor parts was obviously enhanced 4 h after Aza-C NP injection. The signal intensity continues to increase over time and reaches a peak after 24 h (Fig. 4A and B). This clearly demonstrated that Aza-C NPs could effectively aggregate at the tumor site through the EPR effect [38]. To further demonstrate that Aza-C NPs could be effectively enriched in the tumor 24 h after injection of the Aza-C NPs, NIR-II PAI experiments were carried out before and after injection for 24 h. When Aza-C NPs were injected for 24 h, the tumor region showed a obviously enhanced NIR-II PA signal compared to that before the injection of Aza-C NPs (Fig. 4C), further indicating that Aza-C NPs could be effectively enriched at the site of the tumor 24 h after injection. Next, in order to comprehensively assess the in vivo distribution characteristics of Aza-C NPs and their metabolism, we performed in vitro imaging analysis of key organs 24 h after injection of Aza-C NPs. As shown in Fig. 4D, the fluorescent signals were mainly enriched in tumor and liver tissues, indicating hepatic metabolism of Aza-C NPs. Besides, the persistent high signal in the tumor region indicated that the Aza-C NPs had excellent tumor retention ability. To demonstrate that the concentration of Aza-C NPs at the tumor site 24 h after injection could achieve effective tumor PTT, photothermal imaging experiments were performed at the tumor site of Balb/c-nu mice by intravenous injection of equal amounts of PBS and Aza-C NPs. After intravenous injection of PBS or Aza-C NPs for 24 h, under the same irradiation condition (808 nm, 0.6 W cm^−2^, 10 min), the temperature at the tumor region of PBS-injected Balb/c-nu mice increased to 37.8 °C, which indicated negligible heat ablation effect to cervical tumor. By contrast, the tumor parts of Balb/c-nu mice injected with Aza-C NPs showed an obvious high temperature of 67.3 °C (Fig. 4E and F). This marked difference confirms that Aza-C NPs have good photothermal properties in Balb/c-nu mice, which are sufficient to kill tumor cells. As demonstrated by NIR-II FLI/PAI and photothermal imaging of mice, Aza-C NPs could be efficiently enriched at the tumor site of Balb/c-nu mice and subsequently metabolized out of the body by the liver. The enriched Aza-C NPs demonstrated the capacity to execute efficient PTT in Balb/c-nu mice.

**Fig. 5.**
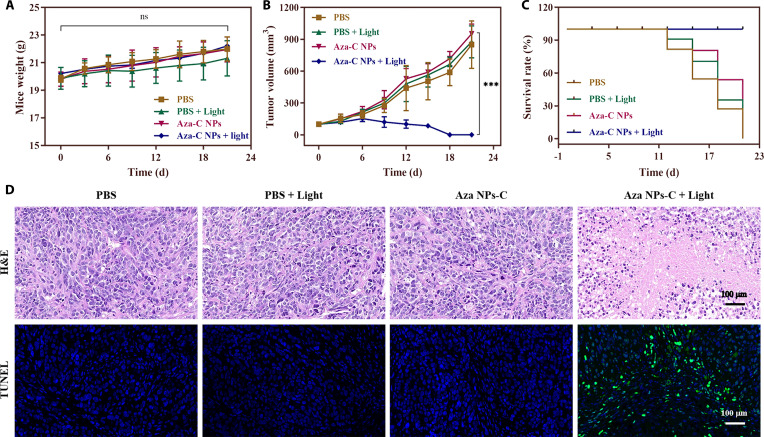
Antitumor efficacy of Aza-C NPs in a Balb/c-nu mice model. (A) Body weight of Balb/c-nu mice in different treatment groups during the treatment period. (B) Tumor growth curves of different treatment groups over 21 d. (C) Survival rates of Balb/c-nu mice in different treatment groups during the treatment period. (D) Representative H&E staining and TUNEL staining analysis of tumor tissues after different treatments. Scale bars, 100 μm. ***P < 0.001.

Based on the excellent enrichment effect and super heat generation performance of Aza-C NPs, we then systematically evaluated their PTT efficacy to cervical tumor. The Balb/c-nu mice with subcutaneous Hela tumors were divided into 4 groups at random. (I) PBS (pH 7.4), (II) PBS + light (808 nm, 0.6 W cm^−2^, 10 min), (III) Aza-C NPs (200 μM), and (IV) Aza-C NPs + light. Body weight showed a consistent trend of changes in all Balb/c-nu mice during treatment (Fig. 5A), confirming the good biosafety of Aza-C NPs. As shown in Fig. 5B and Figs. S12 and S13, the tumor volume of Balb/c-nu mice in groups I to III gradually increased, and the final mouse volume was 7 to 11 times of the initial one. In contrast, the tumor growth of group IV was inhibited after the first treatment, and the tumors almost completely regressed after the second treatment. This phenomenon fully demonstrated the powerful antitumor effect of Aza-C NPs. Furthermore, the survival rates of Balb/c-nu mice in groups I, II, and III began to decrease after 11 d of treatment, and eventually none of them survived. In contrast, 100% of Balb/c-nu mice in group IV survived after the final PTT (Fig. 5C), suggesting that Aza-C NPs combined with light irradiation can effectively improve the survival rate of Balb/c-nu mice. Histopathological analysis showed that, after 21 d of treatment, no obvious pathological changes were detected in hematoxylin and eosin (H&E) staining of the major organs of Balb/c-nu mice in all groups (Fig. S14), indicating that Aza-C NPs are very safe for Balb/c-nu mice. To further assess the treatment effect, systematic histopathological analysis of tumor tissues in each experimental group was performed on day 7 after treatment. H&E staining showed that tumor tissues in groups I to III all maintained relatively intact histological structures, whereas group IV showed obvious pathological changes, manifested as extensive areas of coagulative necrosis accompanied by obvious apoptotic features, such as nuclear consolidation and nuclear fragmentation (Fig. 5D), and the results of the terminal deoxynucleotidyl transferase-mediated deoxyuridine triphosphate nick end labeling (TUNEL) assay were consistent with those of the H&E staining results. These results confirmed that Aza-C NPs combined with light irradiation could effectively kill tumor cells. The results of the biochemical blood analysis showed no marked differences between the experimental groups (Fig. S15), further verifying the systemic safety of Aza-C NPs. In summary, Aza-C NPs with good biosafety exhibit excellent PTT effects, indicating that Aza-C NPs have potential for clinical applications.

## Conclusion

**Fig. 6.**
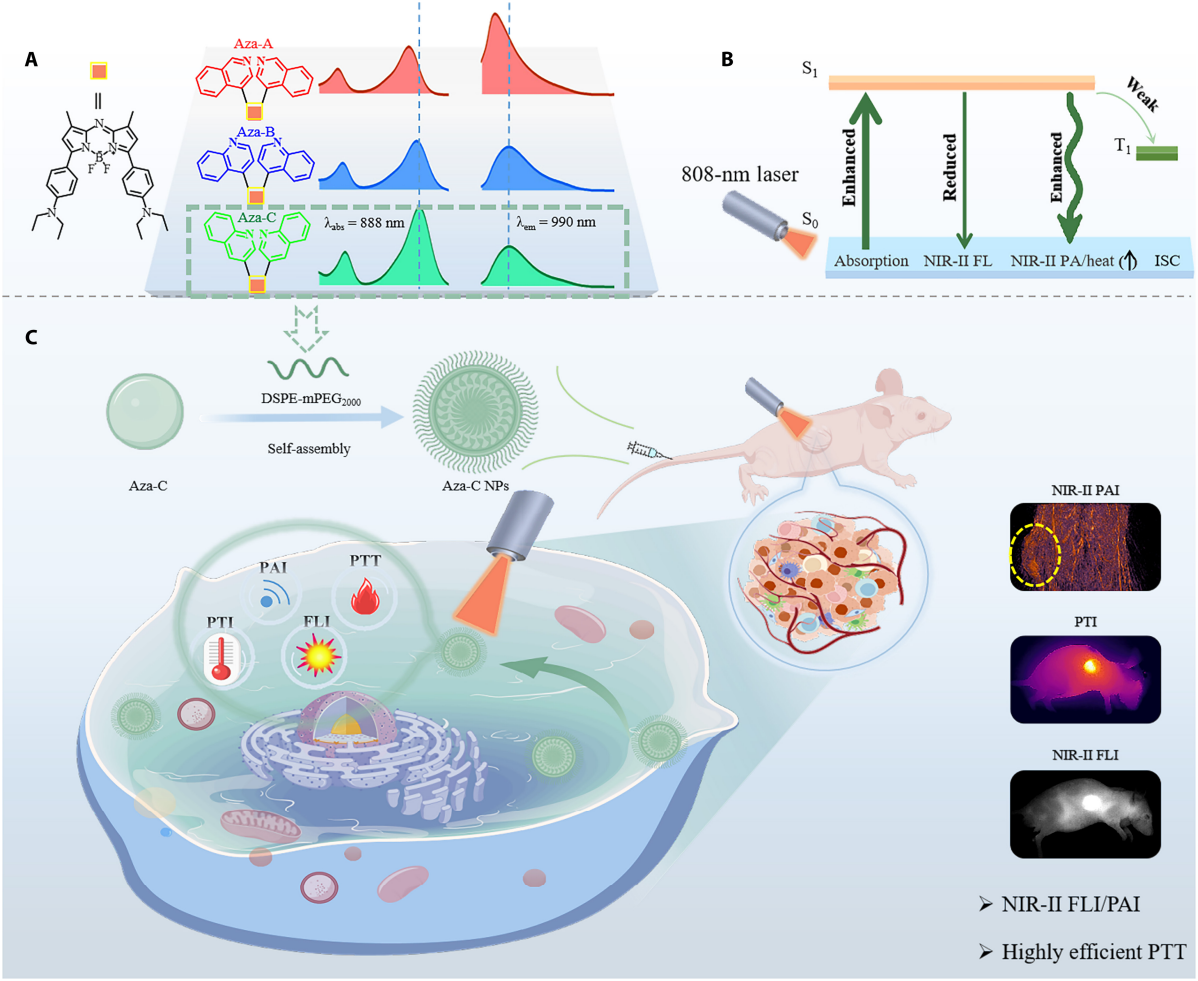
The Aza-C NPs for NIR-II FLI/PAI-guided PTT. (A) Chemical structure and corresponding absorption/emission spectra of Aza-A/B/C dyes. (B) Jablonski diagram describing the enhanced heat production mechanism of Aza-C. (C) In vivo NIR-II FLI/PAI-guided PTT.

In this study, a series of Aza-BODIPY dyes with a D–A configuration (Aza-A/B/C) have been developed through the synergistic integration of diethylaminobenzene, a robust electron donor, (iso)quinoline unit, which functions as electron acceptors, and the Aza-BODIPY core (Fig. 6A). They showed NIR-II absorption and emission, and excellent heat generation capacities. Among them, Aza-C showed optimal heat production performance owing to it strong NIR light capture performance and poor NIR-II emission, as well as weak ROS production capacities (Fig. 6B). For biological application, the self-assembly of DSPE-mPEG_2000_ and Aza-C forming Aza-C NPs exhibited excellent structural stability and biocompatibility as well as concentration-dependent linear FL/PA signals for high-contrast NIR-II FLI/PAI, and obvious tumor suppression (>90%) was achieved by Aza-C NPs under 808-nm light irradiation in a Balb/c-nu mouse model without systemic toxicity (Fig. 6C). In this work, the integration of diagnostic imaging and therapeutic function units into molecular systems has paved the way for novel multifunctional phototherapeutic agents. Besides, the integration of artificial intelligence and deep learning holds important promise to rapidly and efficiently develop NIR-II diagnostic and therapeutic agents for satisfactory tumor therapy [39–41].

## Data Availability

All data are available in the main text or the Supplementary Materials.
