# Supplementary material for: (Iso)quinoline-Modified Aza-Boron-Dipyrromethenes Near-Infrared-II Fluorescence/Photoacoustic Nanotheranostics for Cervical Tumor Photothermal Therapy
Source: Biomater Res. 2025 Dec 9;29:0298. doi: 10.34133/bmr.0298 (PMC12688475; doi:10.34133/bmr.0298)
Supplement: Supplementary 1 — Figs. S1 to S15 Table S1 [file bmr.0298.f1.docx]

**Supporting Information**

**(Iso)quinoline-Modified Aza-Boron-Dipyrromethenes Near-Infrared-II Fluorescence/Photoacoustic Nanotheranostics for Cervical Tumor Phototermal Therapy**

Kexin Wang^1,2^, Zhen Wang^1,2^, Jianfeng Qiu^1,2*^, and Yunjian Xu^1,2*^

1. School of Radiology Shandong First Medical University & Shandong Academy of Medical Sciences, Taian, 271000, China.

2. Medical Science and Technology Innovation Center Shandong First Medical University & Shandong Academy of Medical Sciences, Jinan, 250117, China.

Corresponding author

Jianfeng Qiu, jfqiu@sdfmu.edu.cn

Yunjian Xu, xuyunjian@sdfmu.edu.cn**General information**

Unless otherwise specified, all organic solvents and chemicals were analytical grade reagents obtained from Shanghai Titan Technology Co. and used without further purification. Nuclear magnetic resonance (NMR) spectra were acquired on a Varian Mercury 400 MHz spectrometer for structural verification. Ultraviolet-visible (UV-Vis) absorption spectra were recorded on a Cintra 2020 spectrophotometer, while fluorescence emission spectra were measured using a Horiba Fluoromax-4 spectrofluorometer. **Aza-C NPs** were morphologically characterized by transmission electron microscopy (HT7700, TEM) with an accelerating voltage of 100 KV, and their hydrodynamic diameters were determined via DLS using a Nano ZS90 system. The 808 nm laser was purchased from New Industry Optoelectronics Technology Co. Cytotoxicity testing was performed on a BioTek enzyme labeler. Confocal FLI was conducted on an EVOS M7000 automated 3D digital confocal microscope. In vivo mouse NIR-II FLI experiments were performed on a NIR-II imaging system manufactured by Grand Imaging Technology Co. All in vivo mouse NIR-II PAI experiments were performed using a LOIS-3D/LOIS-3D Plus model NIR-II small animal whole-body 3D PAI system from TomoWave Laboratories. Photothermal imaging was performed with a near-infrared thermal camera (InfiRa AT61U), and light power densities were calibrated using a VLP-2000 power meter.

**Synthesis of Aza-A/B/C and Aza-C NPs dyes**

**Synthesis of 1 - 1**

NaOH solution (1.00 g in 2 mL H_2_O) was added to a solution of isoquinolinaldehyde (1.57 g, 10 mmol) and diethylaminophenone (1.91 g, 10 mmol) in EtOH (15 mL). The mixture was reacted at room temperature for 36 h. Subsequently, 10 mL of deionized water was added and stirred for 2 h. The mixture was filtered, washed with aqueous ethanol and dried. An orange solid product (2.3 g, 71%) was obtained. ^1^H NMR (400 MHz, CDCl_3_) δ 8.96 (d, J = 4.5 Hz, 1H), 8.48 (d, J = 15.5 Hz, 1H), 8.26 (d, J = 8.5 Hz, 1H), 8.17 - 8.15(m, 1H), 8.05 - 8.00 (m, 2H), 7.79 - 7.74 (m, 2H), 7.66 - 7.61 (m, 2H), 6.79 - 6.65 (m, 2H), 3.46 (q, J = 7.1 Hz, 4H), 1.24 (t, J = 7.1 Hz, 6H).

The synthesis of **2/3 - 1** referring to that of **1 - 1**.

Compound **2 - 1** (2.2 g, 66%): ^1^H NMR (400 MHz, CDCl_3_) δ 9.19 (s, 1H), 8.72 (s, 1H), 8.30 - 8.26 (m, 1H), 8.18 (d, J = 8.6 Hz, 1H), 8.01 (d, J = 7.3 Hz, 1H), 7.90 - 7.86 (m, 2H), 7.81 - 7.77 (m, 1H), 7.65 - 7.60 (m, 2H), 6.59 - 6.53 (m, 2H), 3.31 (q, J = 7.1 Hz, 4H), 1.08 (t, J = 7.1 Hz, 6H).

Compound **3 - 1** (2.5 g, 75%): ^1^H NMR (400 MHz, CDCl_3_) δ 9.22 (d, J = 2.2 Hz, 1H), 8.31 (s, 1H), 8.13 (d, J = 8.5 Hz, 1H), 8.05 - 8.02 (m, 2H), 7.93 (d, J = 15.8 Hz, 1H), 7.87 (d, J = 8.2 Hz, 1H), 7.81 (d, J = 15.8 Hz, 1H), 7.77 - 7.73 (m, 1H), 7.61 - 7.57 (m, 1H), 6.73 - 6.68 (m, 2H), 3.46 (q, J = 7.1 Hz, 4H), 1.24 (t, J = 7.2 Hz, 6H).

**Synthesis of 1 - 2**

Compound **1 - 1** (2.0 g, 5 mmol), nitromethane (10 ml), and diethylamine (10 ml) were dissolved in EtOH (50 ml). The mixture was then subjected to reflux for a period of 24 h. Following a period of cooling to room temperature, the resulting mixture was subjected to evaporation to yield a dry substance, which was subsequently dissolved in CH_2_Cl_2_. The organic mixture was then washed with water and brine, dried over sodium sulfate, and concentrated to yield the crude product. The purification process facilitated by column chromatography (EA: PE = 1:4), yielded the desired product **1 - 2**, a grayish-white solid (1.5 g, 78%). ^1^H NMR (400 MHz, CDCl_3_) δ 8.89 (s, 1H), 8.30 - 8.22 (m, 2H), 7.83 - 7.76 (m, 3H), 7.75 - 7.60 (m, 1H), 7.41 (s, 1H), 6.59 (d, J = 9.0 Hz, 2H), 5.22 - 5.15 (m, 1H), 5.03 (dd, J = 13.0, 6.1 Hz, 1H), 4.92 (dd, J = 13.1, 7.9 Hz, 1H), 3.48 - 3.37 (m, 6H), 1.19 (t, J = 7.1 Hz, 6H).

The synthesis of **2/3 - 2** referring to that of **1 - 2**.

Compound **2 - 2** (1.6 g, 82%): ^1^H NMR NMR (400 MHz, CDCl_3_) δ 9.37 (s, 1H), 8.69 (s, 1H), 8.53 (d, J = 8.6 Hz, 1H), 8.27 (d, J = 8.1 Hz, 1H), 8.14 (t, J = 7.2 Hz, 1H), 7.99 - 7.85 (m, 1H), 7.79 (d, J = 9.0 Hz, 2H), 6.61 (d, J = 8.9 Hz, 2H), 5.25 - 5.19 (m, 1H), 5.02 - 4.93 (m, 2H), 3.64 - 3.55 (m, 2H), 3.42 (q, J = 7.0 Hz, 4H), 1.20 (t, J = 7.1 Hz, 6H).

Compound **3 - 2** (1.4 g, 73%): ^1^H NMR (400 MHz, CDCl_3_) δ 8.91 (d, J = 2.4 Hz, 1H), 8.11 - 8.06 (m, 2H), 7.82 - 7.77 (m, 3H), 7.73 - 7.68 (m, 1H), 7.60 - 7.41 (m, 1H), 6.62 - 6.57 (m, 2H), 4.98 (dd, J = 12.9, 6.0 Hz, 1H), 4.83 (dd, J = 12.9, 8.6 Hz, 1H), 4.48 - 4.41 (m, 1H), 3.46 - 3.38 (m, 6H), 1.19 (t, J = 7.1 Hz, 6H).

**Synthesis of Aza-A**

**1 - 2** (1.2 g, 3 mmol) and ammonium acetate (30 g) were dissolved in n-butanol (25 mL). Then, the mixture was heated (150 ^o^C) for 12 h. And the resulting solid was collected and washed with EtOH. Then, the dried solid was dissolved into CH_2_Cl_2_ (60 mL). N,N-diisopropylethane (5 mL) and Et_2_O BF_3_ (5 mL) were added in turn in 10 min. The mixture was left to stir for 6 h at room temperature. The resulting reaction was diluted with dichloromethane and washed with water and brine. The organic layer was dried over magnesium sulfate, and the solvent was removed using low pressure. **Aza-A** (0.14 g) was purified used chromatography on silica gel with CH_2_Cl_2_. ^1^H NMR (400 MHz, DMSO) δ 8.75 (d, J = 4.5 Hz, 2H), 8.40 - 8.21 (m, 6H), 8.03 (d, J = 10.1 Hz, 2H), 7.92 - 7.71 (m, 2H), 7.70 - 7.62 (m, 6H), 6.89 (d, J = 9.4 Hz, 4H), 3.54 (q, J = 7.0 Hz, 8H), 1.20 (t, J = 7.0 Hz, 12H).

The synthesis of **Aza-B/C** referring to that of **Aza-A**.

Compound **Aza-B** (0.31 g): ^1^H NMR (400 MHz, CDCl_3_) δ 9.05 (s, 2H), 8.55 (s, 2H), 8.17 - 8.11 (m, 6H), 7.88 - 7.85 (m, 2H), 7.62 - 7.57 (m, 2H), 7.55 - 7.51 (m, 2H), 7.11 (s, 2H), 6.75 - 6.70 (m, 4H), 3.41 (q, J = 7.1 Hz, 8H), 1.18 (d, J = 7.1 Hz, 12H).

Compound **Aza-C** (0.23 g): ^1^H NMR (400 MHz, CDCl_3_) δ 9.45 (d, J = 2.2 Hz, 2H), 8.99 - 8.96 (m, 2H), 8.21 - 8.13 (m, 6H), 7.77 - 7.70 (m, 4H), 7.53 - 7.49 (m, 2H), 7.32 (s, 2H), 6.79 - 6.73 (m, 4H), 3.45 (q, J = 7.1 Hz, 8H), 1.23 (t, J = 7.1 Hz, 12H).

**Synthesis of Aza-C NPs**

A DSPE-mPEG_2000_ solution (20 mg in 8 mL deionized water) was prepared by sonication (120 W, 2 min). Separately, 1.5 mg of **Aza-C** was dissolved in 4 mL THF and rapidly added dropwise to the DSPE-mPEG_2000_ solution under continuous sonication (120 W, 2 min). The mixture was stirred overnight at 50 °C under argon atmosphere. Purification was achieved via three cycles of PBS (pH = 7.4) washing using a centrifugal filter. The final concentrated solution was utilized in subsequent experiments.

**The loading rate of Aza-C in Aza-C NPs**

The maximum absorption values of **Aza-C** at different concentrations in DMF were detected. According to its concentration dependent absorption value at 888 nm, a standard curve was drawn.

Take 1 mL of **Aza-C NPs** aqueous solution and place it in a 30 kDa molecular weight cut-off ultrafiltration centrifuge device. Centrifuge at 8000 r/min for 20 min at 4 ℃. Collect the concentrated solution and perform freeze-drying to remove the aqueous solvent. Dissolve the dried **Aza-C NPs** in 1 mL DMF, take 100 μL of the solution, further dilute with 2 mL DMF, and measure its absorbance. Quantify the concentration of **Aza-C** based on the above standard curve.

The loading rate of **Aza-C** in **Aza-C NPs** was calculated using below Equation:

**Aza-C** loading (AL) rate = (W_free_/W_total_) × 100% (1)

W_total_: Total quality of DSPE-mPEG_2000_ and **Aza-C**.

W_free_: Quality of **Aza-C**.

**Theoretical calculation of density functional theory (DFT)**

Theoretical calculations were performed using DFT at the B3LYP/6-31G(d) level, and the results showed that all three molecules exhibited electron transfer excitation from 4-diethylaminophenyl to Aza-BODIPY core during the process from S_0_ excitation to S_1_. As shown in Fig. S5, the HOMO-LUMO bandgap of **Aza-A** (1.88 eV) is wider than that of **Aza-B** (1.87 eV) and **Aza-C** (1.80 eV). Subsequently, the molecule was divided into three fragments: fragment 1 was (iso)quinoline, fragment 2 was Aza-BODIPY core, and fragment 3 was 4-diethylaminophenyl (Fig. S6). According to IFCT analysis, it can be seen that compared with **Aza-A/B**, the 4-diethylaminophenyl group of **Aza-C** transfers the least number of electrons to the Aza-BODIPY core, only about 40.33%, while **Aza-A** has 44.21% and **Aza-B** has 46.31%. The proportion of electron transfer involving (iso)quinoline is relatively small, with **Aza-A**: 13.60%, **Aza-B**: 10.59%, **Aza-C**: 20.39%. This result indicates that **Aza-C** has the lowest intramolecular charge transfer (ICT) effect, which suppresses the ICT effect and reduces fluorescence quenching caused by dipole dipole interactions, thereby helping to improve fluorescence emission in polar environments.

**Supplementary figures and table.**


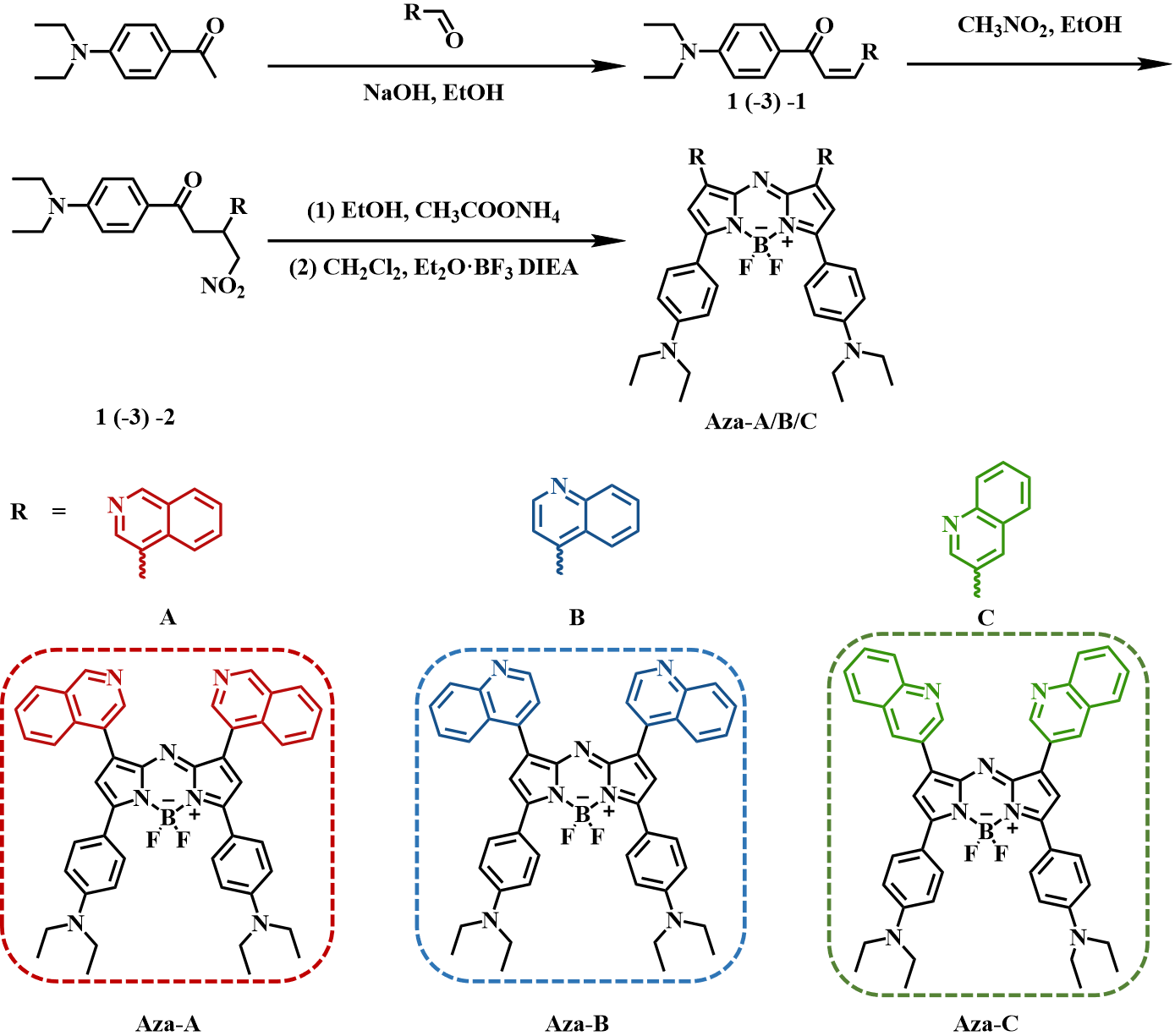


**Fig. S1** The structures and synthetic route of **Aza-A/B/C** dyes.


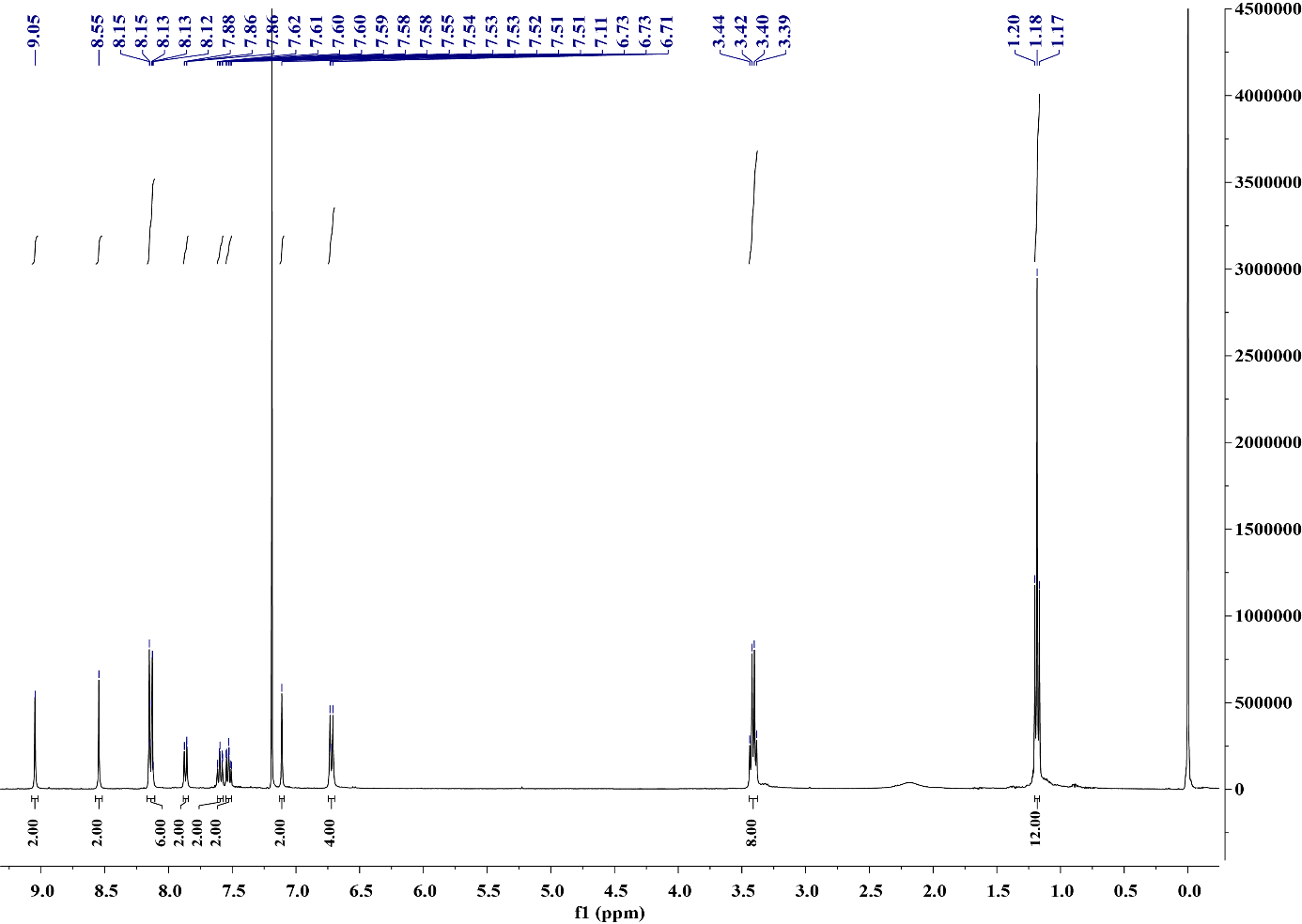


**Fig. S2** ^1^H NMR spectrum of **Aza-A**.

**Fig. S3** ^1^H NMR spectrum of **Aza-B**.

**Fig. S4** ^1^H NMR spectrum of **Aza-C**.

**
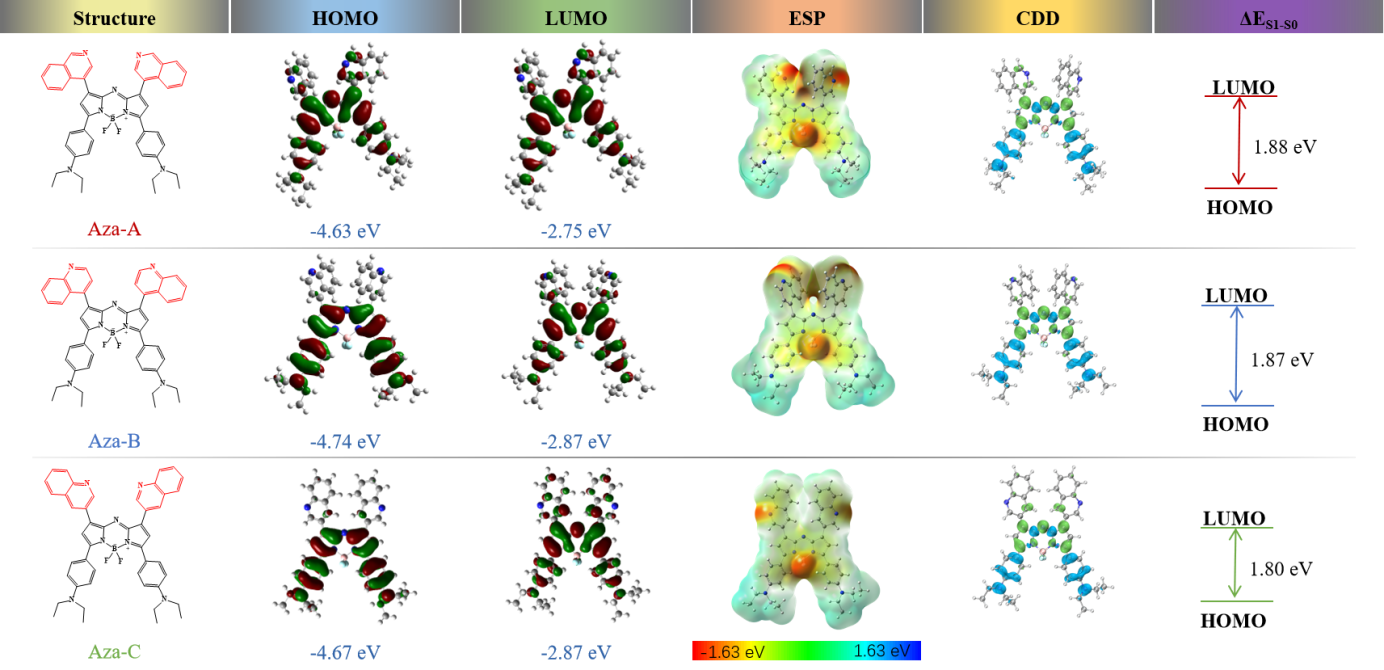
**

**Fig. S5** Theoretical simulations of the **Aza-A/B/C** molecule. The donor-acceptor structures of the **Aza-A/B/C** molecule and corresponding calculations of HOMOs/LUMOs, electrostatic potential (ESP), charge density difference (CDD), and energy gaps in S_1_-S_0_. Interfragment charge transfer analysis.

**
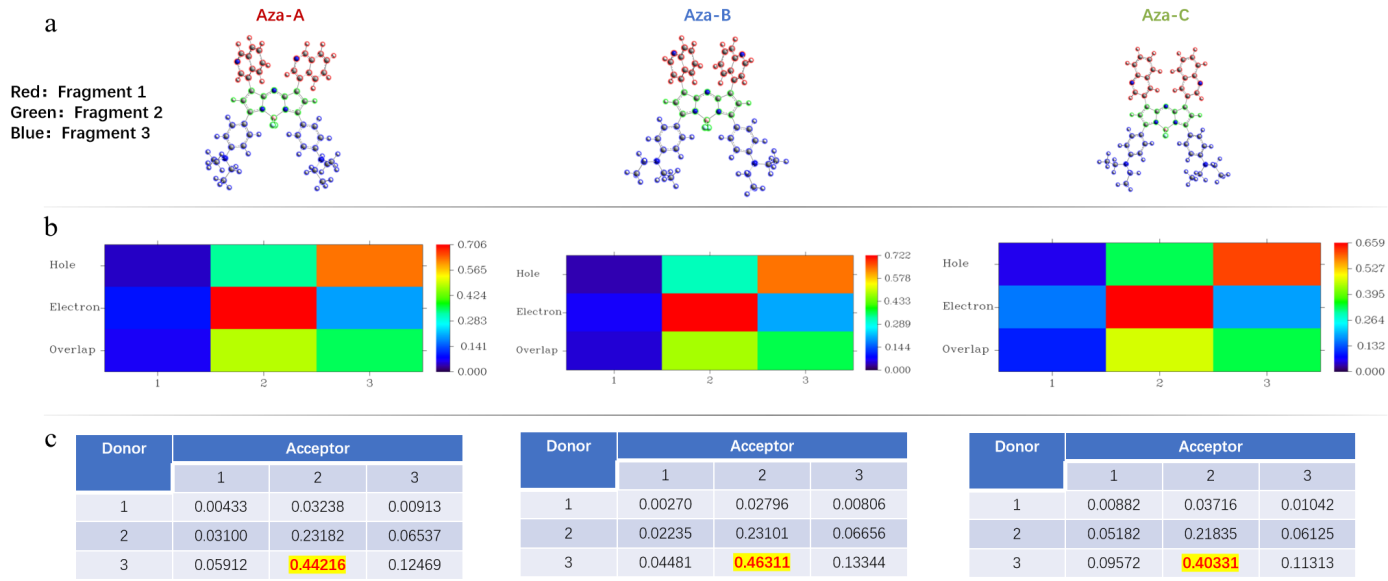
**

**Fig. S6** Interfragment charge transfer. (a) Molecular geometries. (b) Hole and electron distribution. (c) Net charge transfer between fragments.
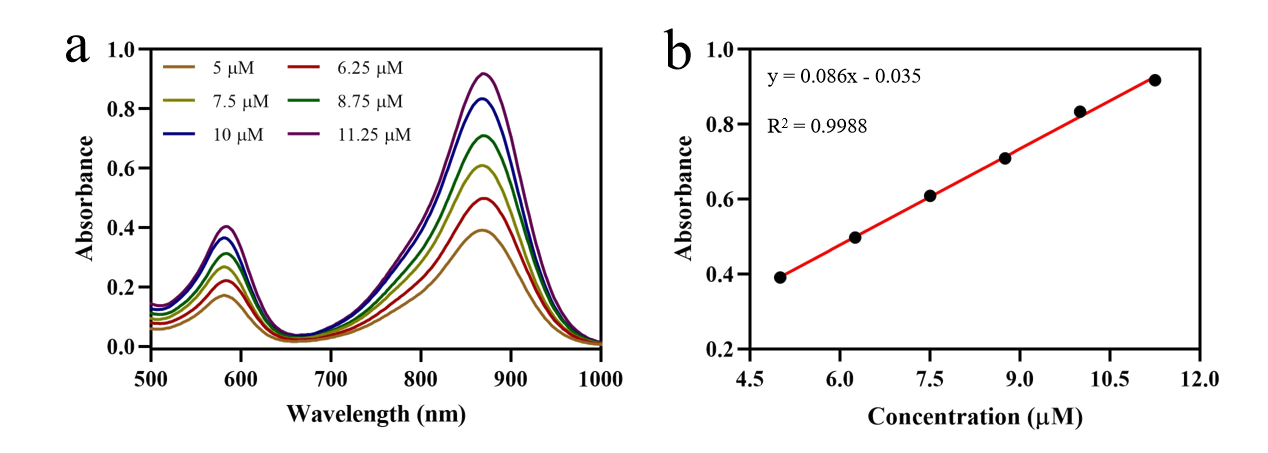


**Fig. S7** (a) Absorption spectra of **Aza-C** and (b) its absorption at 888 nm in different concentrations.

**
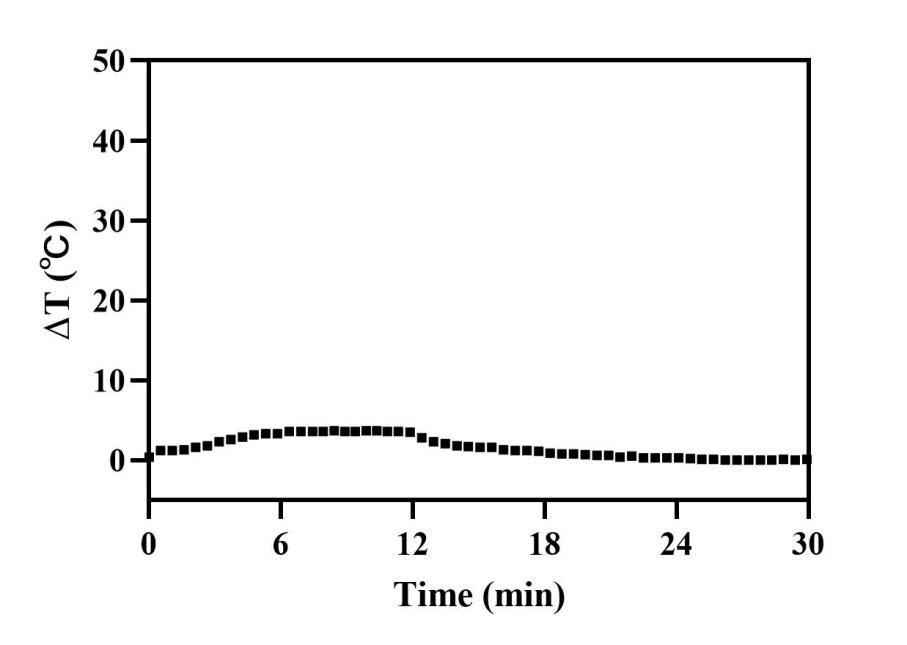
**

**Fig. S8** Heating-cooling curve of water.

**Table S1** Comparison of performance parameters between **Aza-C NPs** and recent NIR-II small molecule dyes.

| Name | Laser Irradiation | Light-to-heat Conversion Efficiency | Fluorescence Quantum Yield | Therapeutic Effect |
| --- | --- | --- | --- | --- |
| **Aza-C NPs** | 808 nm | 58.2% | less than 0.10% | The tumor has been completely ablated. |
| BBTPPRO NPs[20] | 808 nm | 31.19% | Not Specified |  |
| PSiPR[21] | 808 nm | 39.65% | Not Specified |  |
| CNPJ[22] | 808 nm | 49.33% | 2.57% |  |
| TPE-CyA NPs[27] | 1064 nm | 46.27% | 0.99% |  |

**
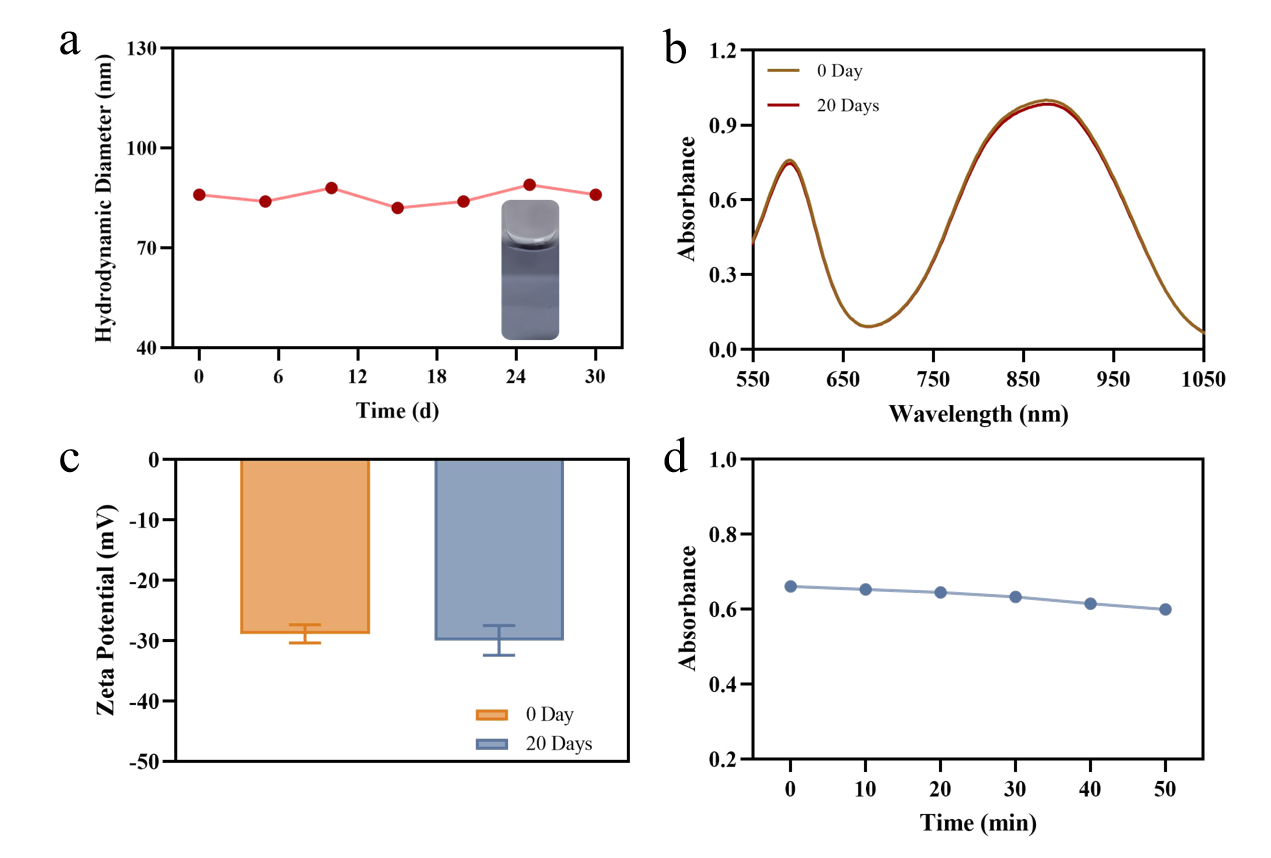
**

**Fig. S9** (a) Hydrodynamic diameter of **Aza-C NPs** during 20 days of storage at room temperature. (b) Absorption spectra and (c) zeta potential of **Aza-C NPs** in PBS solution before and after 20 days of storage. (d) The changes in maximum absorption values of **Aza-C NPs** in serum after continuous irradiation with 808 nm laser for different duration.


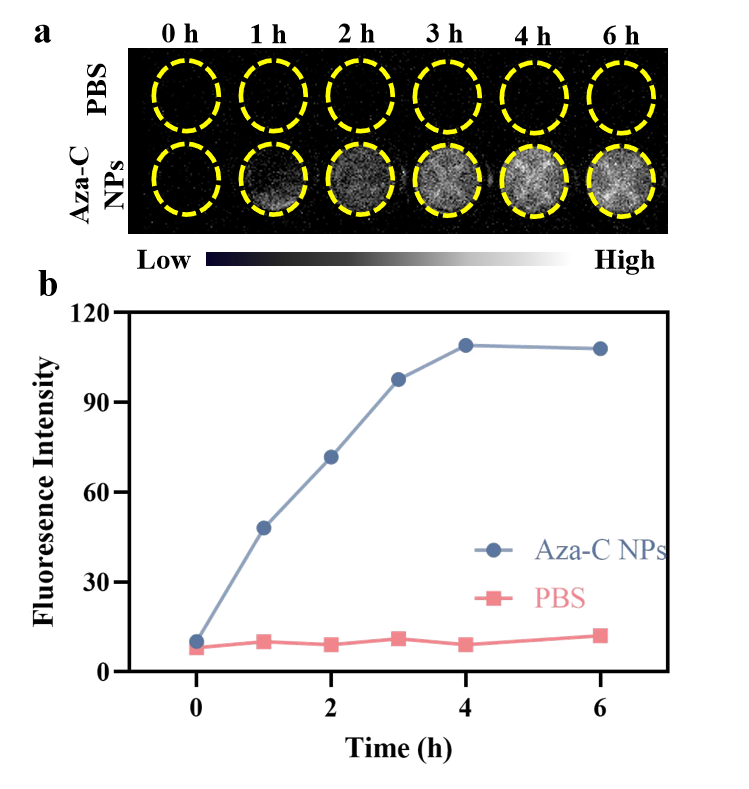


**Fig. S10** (a) NIR-II fluorescence imaging of Hela cells under different time points and (b) fluorescence intensity curves of the cell region over time.


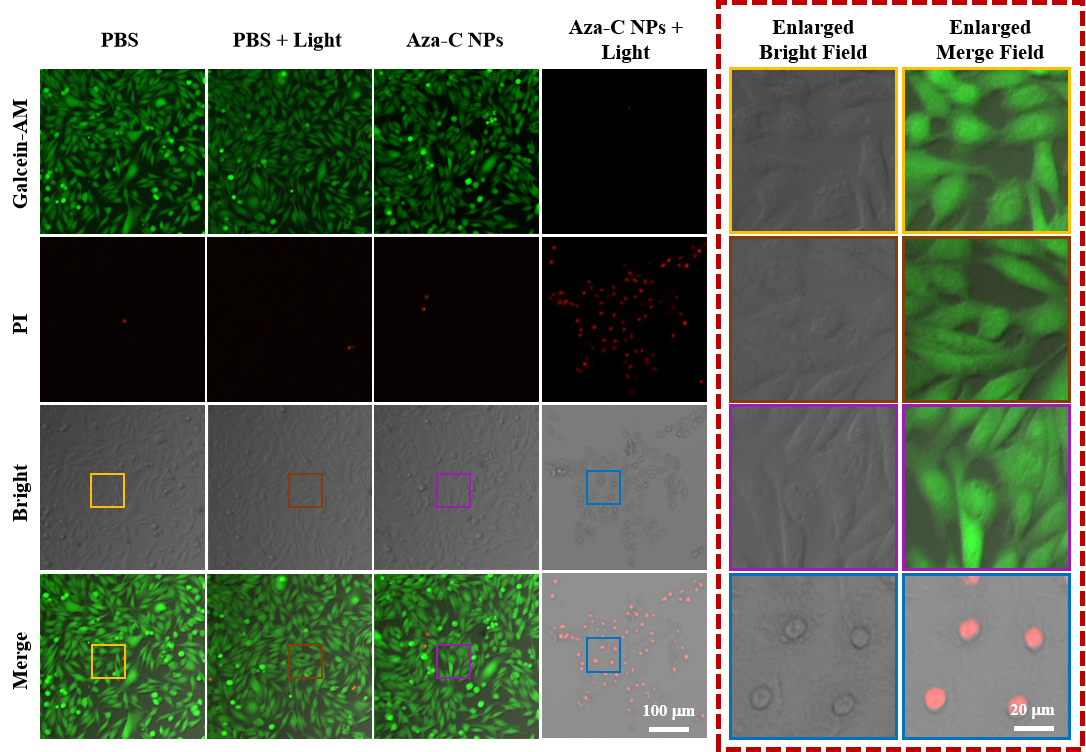


**Fig. S11** Confocal FLI of NIH3T3 cells stained with Calcein-AM (Green, live cells) and PI (red, dead cells) after different treatments. Scale bars: 100 μm. The dashed boxes provide magnified views of the corresponding regions of interest from the left image, with a scale bar of 20 μm.

**
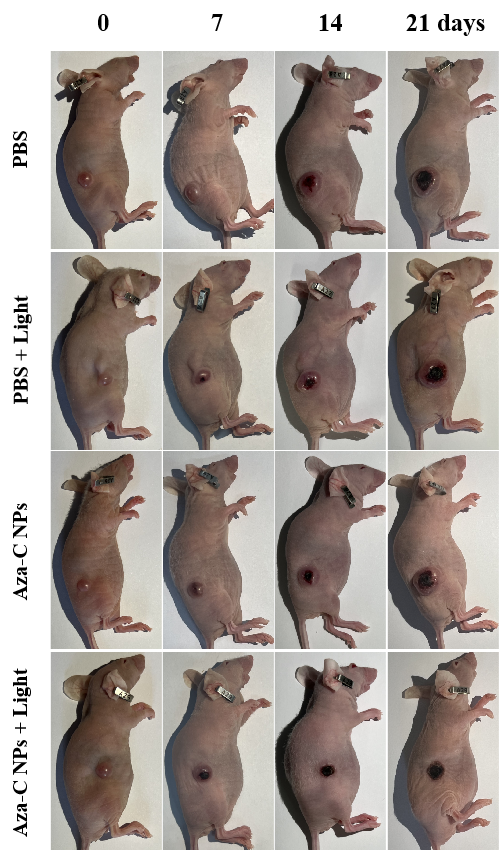
**

**Fig. S12** Photos of mice in different treatment groups at different periods of time.


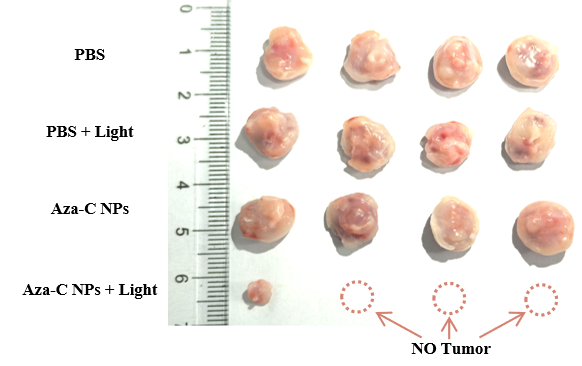


**Fig. S13** Photos of tumor size at day 21 in different treatment groups.


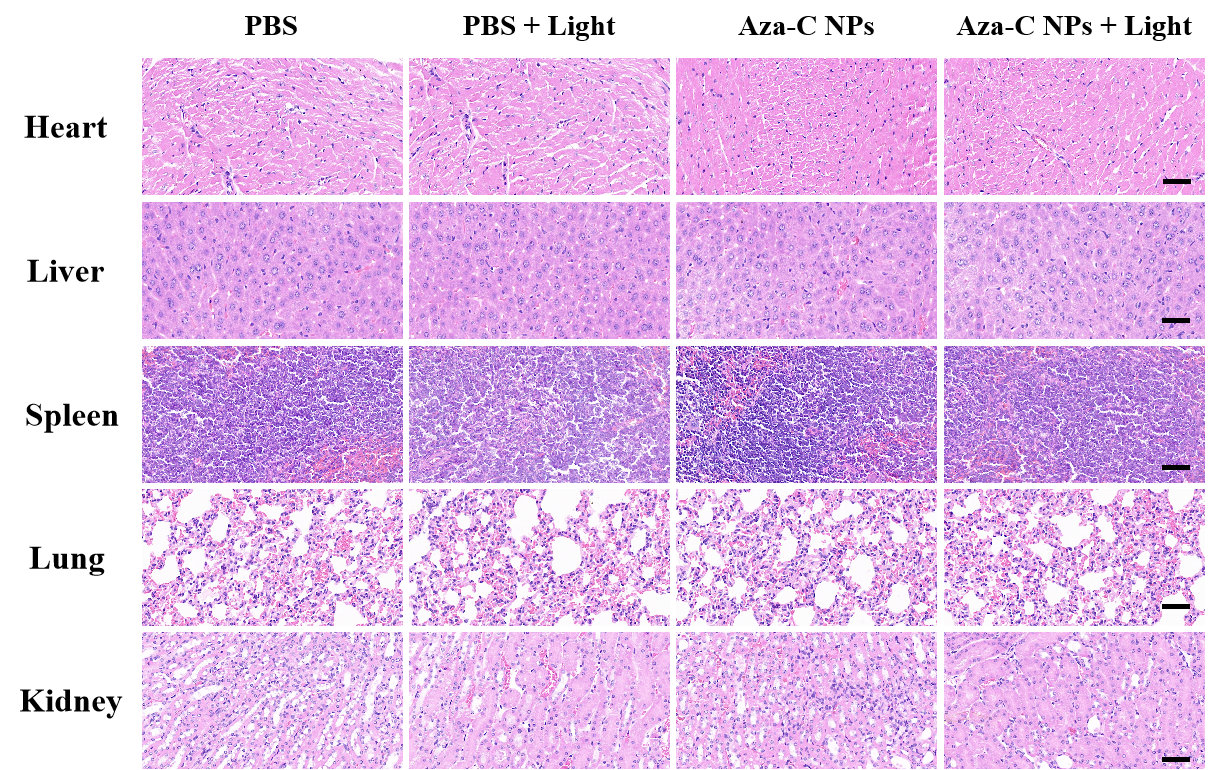


**Fig. S14** H&E staining of major organs excised from different treatment groups. Scale bars: 100 µm.
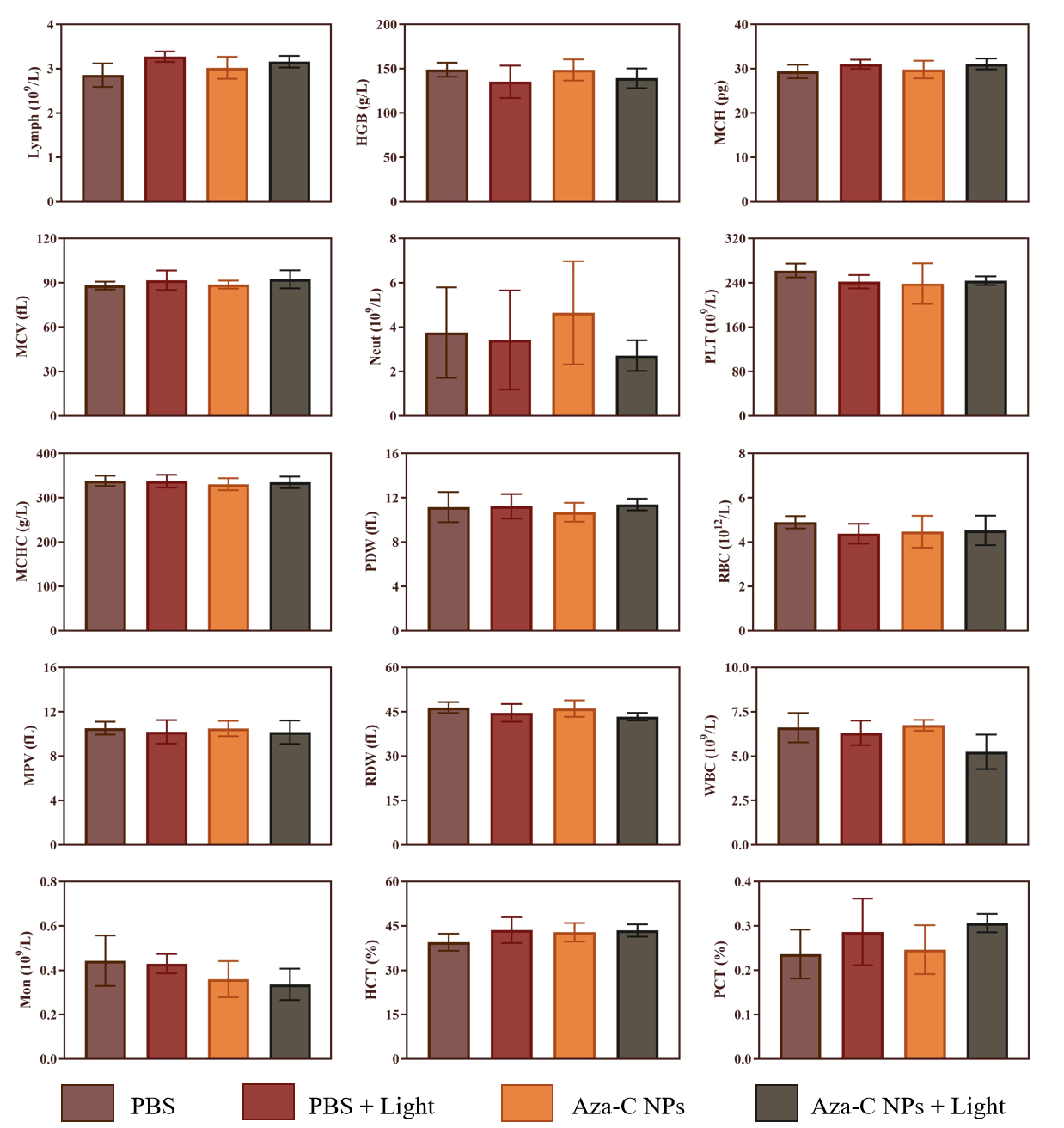


**Fig. S15** Complete blood panel analysis of different treatment groups (3 mice per group). Data are presented as mean ± standard deviation.
